# SBRT of ventricular tachycardia using 4pi optimized trajectories

**DOI:** 10.1002/acm2.13454

**Published:** 2021-10-22

**Authors:** Cristiano Q. M. Reis, Brian Little, Robert Lee MacDonald, Alasdair Syme, Christopher G. Thomas, James L. Robar

**Affiliations:** ^1^ Department of Radiation Oncology Dalhousie University Halifax Nova Scotia Canada; ^2^ Department of Medical Physics Scotia Health Authority, Nova Halifax Nova Scotia Canada; ^3^ Department of Physics and Atmospheric Science Dalhousie University Halifax Nova Scotia Canada; ^4^ Beatrice Hunter Cancer Research Institute Halifax Nova Scotia Canada; ^5^ Department of Radiology Dalhousie University Halifax Nova Scotia Canada; ^6^ Department of Radiation Oncology, London Regional Cancer Program London Health Sciences Centre 790 Commissioners Road East London ON N6A 4L6 Canada; ^7^ Adaptiiv Medical Technologies Inc 405‐1344 Summer Street Halifax, NS B3H 0A8 Canada

**Keywords:** 4pi trajectories, radiosurgery, SBRT, ventricular tachycardia

## Abstract

**Purpose:**

To investigate the possible advantages of using 4pi‐optimized arc trajectories in stereotactic body radiation therapy of ventricular tachycardia (VT‐SBRT) to minimize exposure of healthy tissues.

**Methods and materials:**

Thorax computed tomography (CT) data for 15 patients were used for contouring organs at risk (OARs) and defining realistic planning target volumes (PTVs). A conventional trajectory plan, defined as two full coplanar arcs was compared to an optimized‐trajectory plan provided by a 4pi algorithm that penalizes geometric overlap of PTV and OARs in the beam's‐eye‐view. A single fraction of 25 Gy was prescribed to the PTV in both plans and a comparison of dose sparing to OARs was performed based on comparisons of maximum, mean, and median dose.

**Results:**

A significant average reduction in maximum dose was observed for esophagus (18%), spinal cord (26%), and trachea (22%) when using 4pi‐optimized trajectories. Mean doses were also found to decrease for esophagus (19%), spinal cord (33%), skin (18%), liver (59%), lungs (19%), trachea (43%), aorta (11%), inferior vena cava (25%), superior vena cava (33%), and pulmonary trunk (26%). A median dose reduction was observed for esophagus (40%), spinal cord (48%), skin (36%), liver (72%), lungs (41%), stomach (45%), trachea (53%), aorta (45%), superior vena cava (38%), pulmonary veins (32%), and pulmonary trunk (39%). No significant difference was observed for maximum dose (*p* = 0.650) and homogeneity index (*p* = 0.156) for the PTV. Average values of conformity number were 0.86 ± 0.05 and 0.77 ± 0.09 for the conventional and 4pi optimized plans respectively.

**Conclusions:**

4pi optimized trajectories provided significant reduction to mean and median doses to cardiac structures close to the target but did not decrease maximum dose. Significant improvement in maximum, mean and median doses for noncardiac OARs makes 4pi optimized trajectories a suitable delivery technique for treating VT.

## 1 INTRODUCTION

Ventricular tachycardia (VT) is a heart arrhythmia characterized by a fast and abnormal cardiac rhythm due to irregular electrical signals in the lower chambers of the heart. For some patients, failure to manage the accelerated rhythm may lead to sudden cardiac death.^1^ Traditional treatments for VT often include the use of an implantable cardioverter defibrillator (ICD)^2^, ^3^; use of radiofrequency ablation by feeding a catheter usually into the femoral artery to access the heart^4^, ^5^, ^6^; or use of antiarrhythmic medications.^7^ However, these techniques achieve limited success, for example, with catheter ablation involving a recurrence rate of approximately 50%,^8^, ^9^, ^10^, ^11^ likely due to an incapacity to ablate critical parts of the substrate of the cardiac arrhythmia.^12^ Moreover, the early mortality rate can be approximately 5% within the first 31 days following radiofrequency ablation.^13^

These limitations have prompted the investigation of the use of single fraction stereotactic body radiation therapy (SBRT) to ablate the substrate of cardiac arrhythmias. The feasibility was first demonstrated with an animal study for treating atrial cardiac arrhythmia using stereotactic robotic radiosurgery (Cyber‐Heart System).^14^ Thereafter, the first VT patient treated with SBRT was reported by Loo et al. in 2015 with a single radiation fraction of 25 Gy.^15^ Recent studies^16^, ^17^, ^18^, ^19^, ^20^ employing SBRT have reported a significant reduction in VT burden without any significant acute toxicity following a single fraction of 25 Gy to the arrhythmogenic scar region of the heart. In this sense, VT‐SBRT has become a promising technique for treating this form of cardiac arrhythmia, offering an additional option to a narrowly‐selected subset of patients.^21^

However, since VT‐SBRT is a novel technique, a range of technical issues still need to be further investigated and optimized.^22^ These include cardiac and respiratory motion management, methods for accurate co‐registration of several image modalities (e.g., computed tomography, magnetic resonance and electrocardiographic imaging maps), target volume delineation, and the optimization of treatment beam geometry to provide maximum sparing of proximal organs‐at‐risk (OARs). VT‐SBRT treatment planning performed to date has relied mainly on dose constraints available in Radiation Therapy Oncology Group reports for other SBRT techniques such as those for lung (RTOG 0915)^23^ and spine (RTOG 0631),^24^ as well as on Task Group Report 101 of the AAPM (TG‐101).^25^ It is clear that treatment planning approaches must emphasize minimization of cardiac radiation toxicity.^26^, ^27^ Recent studies have shown that cardiac structures are highly radiosensitive and since they are usually not readily visualized on treatment planning CTs, the use of combined imaging techniques with high soft tissue contrast is crucial in order to enable cardiac structures sparing in radiation therapy treatment planning.^28^

Commonly, SBRT is performed using a volumetric modulated arc therapy (VMAT) technique, delivering a set of coplanar or noncoplanar treatment arcs. Often the arc geometry is chosen arbitrarily by the treatment planner. Methods of optimizing this geometry in an automated way have been proposed in the literature.^29^, ^30^, ^31^, ^32^, ^33^, ^34^ For example, MacDonald et al. proposed a customizable algorithm in order to generate a navigable ideal trajectory based on the amount of overlap in the beams‐eye‐view (BEV) between the planning target volumes PTVs and OARs, and thus demonstrating the capacity to improve radiation therapy dose distributions in cranial SRT.^33^, ^34^ Their results show an average decrease of 19% and 15% in mean and maximum doses respectively to OARs when compared to conventional treatment trajectories.^33^

It is well recognized that 4pi methodology has already been extensively explored, but mainly for stereotactic techniques for treating brain lesions, to minimize exposure of healthy tissues. Furthermore, stereotactic ablation of ventricular tachycardia presents a geometry of Organs‐at‐Risk (OARs) quite unlike any other anatomical site treated with radiation therapy, and given this, the likely performance of the 4pi approach it is still not clear based on previous experience. This, combined with a paucity of previous systematic study of favorable treatment geometries for VT treatment, compelled us to investigate the use of 4pi‐optimized arc trajectories in SBRT of ventricular tachycardia to minimize exposure to healthy tissues, especially for cardiac structures close to the target.

## 2 METHODS

### 2.1 Clinical data and CT contouring

In this study, computed tomography (CT) datasets of the thorax (voxel size 0.5 mm x 0.7 mm x 0.7 mm) for *N* = 15 patients obtained from a public repository^35^, ^36^ were imported into the treatment planning system (Eclipse v.15.6, Varian Medical Systems, Inc., Palo Alto, USA) for contouring of the main OARs and definition of the PTV. CT datasets were chosen to provide adequate image quality in the vicinity of cardiac structures, including right and left atria, right and left ventricles, pulmonary trunk, pulmonary veins, aorta, superior vena cava (SVC) and inferior vena cava (IVC). Additional OARs were contoured for the purpose of dose sparing, including spinal cord, esophagus, trachea, lungs, liver, stomach and skin.

**FIGURE 1:**
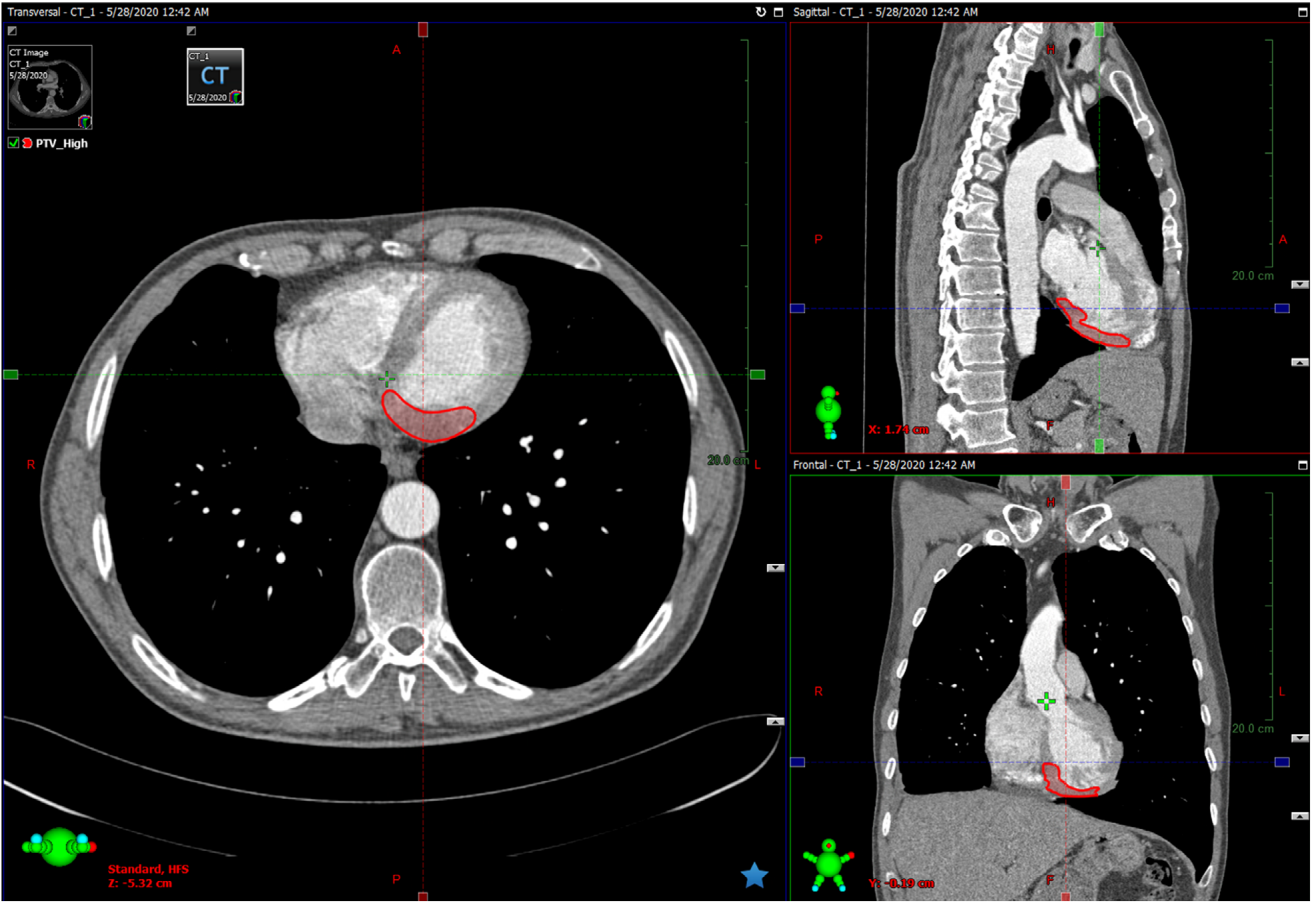
Thorax CT image of one of the patients used in this work for VT‐SBRT planning showing a realistic PTV based on occurrences reported by Neuwirth et al.^19^

Realistic target regions corresponding to VT substrate were defined within the endocardium around the left ventricle based on occurrences reported in the study by Neuwirth et al.^19^ with regard to both location and volume. Figure 1 shows an example of a PTV defined for one of the CT images used in this work. The mean volume of the targets averaged over the 15 patients was 23.2 ± 4.9 cm^3^ which is close to the mean value of 22.2 cm^3^ reported by Neuwirth et al. Similarly to that study, no additional margin was added to the PTVs defined in this work.

### 2.2 Treatment planning

#### 2.2.1 Conventional beam trajectory

As a novel procedure VT‐SBRT has no well‐established delivery technique for treatment planning and delivery. However, a common arc geometry for SBRT (e.g., of spine, prostate, lung, liver) includes two or more partial or full (360°) arcs of the beam. In some cases, nonzero couch angles may also be used to avoid overexposure of critical OARs. Knutson et al.^18^ used two noncoplanar arcs with couch angle of ±15° and one with axial arrangement in their VT‐SBRT study. For this work, the conventional beam trajectory was defined as two coplanar, full arcs with ±30° collimator angles and without couch rotation. Isocenters were placed outside of the target and close to the midline to avoid collision of the gantry with the couch or patient.

#### 2.2.2 4pi optimized beam trajectories

**FIGURE 2:**
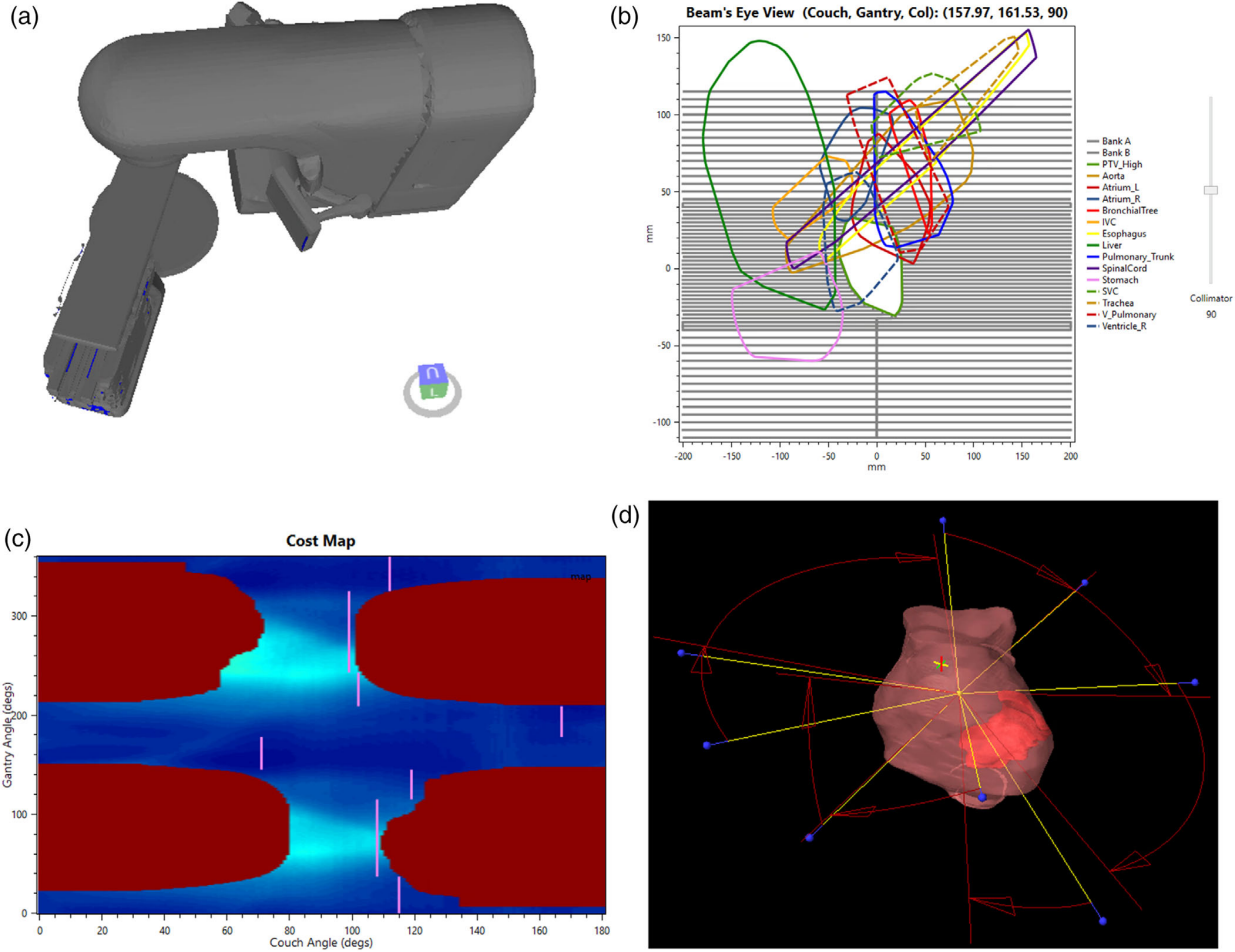
(a) Couch and gantry position corresponding to 292^o^ and 18^o^ eclipse coordinates. (b) Beam's eye view corresponding to couch and gantry position shown in (a). (c) Overlap map for the selected OARs shown in (b) with eight final optimized trajectories indicated as straight lines. Couch and gantry angles shown in the overlap map are defined in the geometric overlap space as defined by MacDonald and Thomas.^33^ Equivalent Eclipse coordinates for the couch are given by: *eclipseCouch* = 90^o^ – *mapCouch*, for *mapCouch* < 90^o^, and *eclipseCouch* = (360^o^ − *mapCouch*) + 90^o^, for 90^o^ ≤ *mapCouch* ≤ 360^o^. Similarly, gantry values in Eclipse are obtained by: *eclipseGantry *= 180^o^ − *mapGantry*, for *mapGantry* < 180^o^, and *ecliplseGantry* = (360^o^ − *mapGantry*) + 180^o^, for *mapGantry* ≥ 180^o^. Darker blue colors are assigned to regions of minimum overlap in opposite to lighter colors for high overlapping. Collision zones are represented as dark maroon patches on the left superior corner (eclipse couch from 19^o^ to 90^o^), on the right inferior corner (eclipse couch from 341^o^ to 270^o^), on the left inferior corner (eclipse couch from 11^o^ to 90^o^) and right superior corner (eclipse couch from 349^o^ to 270^o^) . (d) A 3D model view of the eight VMAT fields planned with the optimized trajectories

The method of a dynamic trajectory‐based couch motion proposed by MacDonald et al.^33^, ^34^ was used in this work for generating treatment arc geometries with optimized trajectories that minimize the geometric overlap between PTV and OARs in the BEV. The 4pi algorithm was implemented using the Eclipse Scripting API (ESAPI), thus allowing for definition of optimized trajectories within the Eclipse treatment planning system. Briefly, the algorithm works by projecting both the PTV and OAR onto a two‐dimensional isocentric plane for every possible gantry and couch coordinate sampled at a resolution of 1°. This resolution is used to balance the efficiency of calculation and the resolution necessary to characterize the geometry. The chosen resolution is finer than the sampling of VMAT arcs in the planning system, which uses 2° per control point. The amount of geometric overlap, the cost, within each BEV is evaluated based upon the projected areas of the structures and ranked with a large amount of overlapping being assigned a high penalty.^33^, ^34^ The overlap information is then filed in a couch‐gantry space (*θ*_C_, *θ*_G_) and the magnitude of the overlap is indicated via a color map (an overlap map). Figure 2(a) shows a particular pair of couch and gantry angles with the respective BEV (Figure 2b) with selected structures projected on the isocentric plane.

Since the OARs have different dose tolerances the overlap ranking for each OAR is weighted according to dose constraints that are configurable by the user. Table 1 presents constraints used in this work in order to calculate weighting factors *w*, defined as 1/*D*_tol_, where *D*_tol_ is the OAR dose tolerance limit in Gy.^33^ Thus, an OAR with a low dose tolerance would represent high cost in the overlap map and those angles would not be favorable for trajectory optimization. For most OARs, maximum dose (*D*_max_) values from Table 1 were used as *D*_tol_ for calculating the weighting factors since that represents a more restrictive parameter for dose sparing. Furthermore, *D*_max_ is the most appropriate parameter to take into account when dealing with serial OARs. It is important to note that the weighting factors as defined by MacDonald and Thomas incorporate tolerance dose values to define the relative importance of OARs when weighting their overlap with the PTV in the cost function. In that sense, they do not have the same meaning as the weighting factors that we usually specify in treatment planning.

**TABLE 1:** Dose constraints for single fraction radiation therapy as recommended in RTOG 0915^24^, RTOG 0631^25^, and TG‐101^26^. Maximum dose *D*_max_ is reported as the dose at the volume of 0.03 cubic centimeters (cc) of the OAR

OAR	Dose constraint	Endpoint (≥ Grade 3)
Spinal cord	*D*_max_ ≤ 14 Gy	Myelitis
	*V*_7Gy_ < 1.2 cc	
	*V*_10Gy_ < 0.35 cc	
Esophagus	*D*_max_ ≤ 15.4 Gy	Stenosis/fistula
	*V*_11.9_ Gy < 5 cc	
	*V*_16Gy_ < 0.03 cc	
Heart/pericardium	*D*_max_ ≤ 22 Gy	Pericarditis
	*V*_16Gy_ < 15 cc	
Great vessels	*D*_max_ ≤ 37 Gy	Aneurism
	*V*_31Gy_ < 10 cc	
Trachea	*D*_max_ ≤ 20.2 Gy	Stenosis/fistula
	*V*_10._5 Gy < 4 cc	
Skin	*D*_max_ ≤ 26 Gy	Ulceration
	*V*_23Gy_ < 10 cc	
Stomach	*D*_max_ ≤ 12.4 Gy	Ulceration/fistula
	*V*_11.2_ Gy < 10 cc	
Lungs	*V*_7Gy_ < 1500 cc	Basic lung functions and pneumonitis
	*V*_7.4 Gy_ < 1000 cc	
Liver	*V*_9.1 Gy_ < 700 cc	Basic liver function

Dose constraints presented in Table 1 are recommended values for one single fraction SBRT for lung (RTOG 0915),^23^ spine (RTOG 0631),^24^ as well as recommendations from TG‐101,^25^ and were also considered in the VMAT optimization. Since cardiac structures constraints are not well established for single fraction treatment, a conservative maximum dose value of 4 Gy was used for all cardiac structures in calculating the weighting factors. That value was estimated based on the dose limitation value of 8.5 Gy to the heart associated with poorer survival of lung cancer patients treated with 55 Gy in 20 fractions reported by McWilliam et al.^26^ An EQD2 value of 5.8 Gy was found for that constraint using the linear‐quadratic (LQ) model with *α*/*β* = 3. The value of 4 Gy was then determined as an extrapolation to a single fraction treatment to provide the same EQD2 value. Despite limitations of using the LQ model to predict cell killing at high dose single fraction, the value obtained is low enough to prioritize cardiac structures in terms of overlap ranking when running the 4pi optimization with the ESAPI. Furthermore, no significant differences were observed in the optimized trajectories when using weighting factor with smaller values of *D*_tol_, for example, 1 Gy, for cardiac structures.

The overlap map can then be calculated as a function of the couch (c) and gantry (g) coordinates according to^33^

(1) $$E\left(c,g\right)=\sum_{i}w_{i}\;\;\times F\times \left[\frac{L_{i}\left(c,g\right)}{A_{t}\left(c,g\right)}\times \frac{L_{i}\left(c,g\right)}{A_{i}\left(c,g\right)}\right]$$

where *w_i_* is the relative weighting factor for the *i*th OAR; the factor *F* was introduced by MacDonald and Thomas^33^ to account for the possibility of the OAR being between the PTV and the source (foreground overlap, *F* = 1) or behind the PTV (background overlap, *F* = 1/10); *A_i_(c,g)* and *A_t_(c,g)* are the areas of the *i*th OAR and the PTV, respectively, projected onto the isocentric plane; and *L_i_(c,g)* is the overlap area between the PTV and the *i*th OAR.

To select trajectories that will be deliverable, collision zones were experimentally determined on a TrueBeam STx linear accelerator (Varian Medical Systems, Inc., Palo Alto, USA) using an anthropomorphic phantom placed on the treatment couch. Patient size may heavily influence the set of permissible gantry or couch angles for treatment planning. A more ambitious goal of the 4pi method would be to calculate patient specific collision zones from data of treatment room and patient CT planning. While this is an active area of development,^37^ it is out of the scope of this project. In the present work, an anthropomorphic solid water phantom with dimensions equivalent to an average patient was used and set up on the couch with a wing board and some cushions to simulate the patient positioned with arms up. Additionally, a margin of 5 cm from the collision points was considered to account for patient size variability. The couch was set to position the mechanical isocentre near the middle of the body. Collision zones were determined by rotating the gantry and couch through their full range of motion and recording the limiting angles at which the collision system was activated. The measured gantry and couch angles were converted into the respective coordinates of the couch‐gantry space (*θ*_C_, *θ*_G_) defined at the isocentric plane referred above and were then provided to the ESAPI application. Figure 2(c) shows an overlap map calculated for one patient and for selected OARs with the final optimized trajectories superimposed as straight lines, and dark maroon patches representing the forbidden collision zones. The ESAPI application provides the final optimized set of allowable minimum‐cost arcs as described by MacDonald et al.^34^ A treatment plan is then created within Eclipse using the set of optimized trajectories (couch and gantry angles) generated by the ESAPI. Figure 2(d) shows the corresponding arcs planned in Eclipse with the optimized trajectories. We note that while, in concept, this 4pi method can determine complex trajectories involving simultaneous couch and gantry motion, this study used an optimized series of arcs with fixed couch angles,^34^ since such a plan would not be deliverable given current limitations of the treatment platform. It is important to recognize that using multiple arcs at multiple couch angles may increase positioning uncertainty. However, we point out that i) other practitioners are already using multiple couch angles for VT ablation, but without systematic background on the selection of angles, ii) positioning uncertainty is probably of the same order of those found in other stereotactic techniques which also make use of noncoplanar fields using different couch rotations such as in cranial SRS, and iii) image guidance systems exist that are able to confirm positioning between couch rotations.

When calculating the optimized trajectories with the ESAPI some OARs were not included since they would overlap with the PTV at every direction. Table 2 shows which OARs were included in the geometrical optimization with their respective weighing factor.

**TABLE 2:** Specification of OARs used in the trajectory optimization with their respective weighting factors (*w* = 1/*D*_tol_)

OARs	Included in the 4pi optimization	Weighting factor
Esophagus	Yes	1/15
Spinal cord	Yes	1/14
Skin	No	–
Liver	Yes	1/9
Lungs	No	–
Stomach	Yes	1/12
Trachea	Yes	1/20
Heart‐PTV	No	–
Right atrium	Yes	1/4
Left atrium	Yes	1/4
Right Ventricle	Yes	1/4
Left Ventricle	No	–
Aorta	Yes	1/4
IVC	Yes	1/4
SVC	Yes	1/4
Pulmonary veins	Yes	1/4
Pulmonary trunk	Yes	1/4

### 2.3 Plan evaluation and data analysis

A conventional (two coplanar arcs) plan and a 4pi‐optimized trajectory plan were created for each patient. A single fraction dose of 25 Gy was prescribed to the PTV, and both plans were normalized to ensure 95% coverage of the PTV volume was covered by 100% of the prescription dose. Consistent with our clinical planning practice, a tuning structure was created with an inner margin of 3 mm from the PTV to keep hotspots (areas receiving doses > 100% of prescription dose) within the target. An additional tuning structure (3 mm ring) was also used to surround the PTV in order to increase dose fall‐off. For both plans, the same VMAT optimization dose objectives for OARs were set, based on dose constraints presented in Table 1. For cardiac structures the conservative value of 4 Gy was used to minimize dose as much as possible. The automatic normal tissue optimization (NTO) was turned on for all plans. The VMAT optimization was run following the same procedure with the optimizer for both plans (conventional and 4pi) in order to allow a consistent comparison between the results obtained with the two techniques. Accordingly, after the optimization was started, the optimizer was paused until reaching stabilization and subsequently allowed to continue with no further interaction, that is, adjustment of objectives or priorities.

The PTV homogeneity index (HI) was calculated and compared for both plans according to^38^:

(2) $$\mathrm{HI}\;=100\%\;\times \frac{D_{5}-D_{95}}{D_{p}}$$

where D_5_ and D_95_ are doses to 5% and 95% of the volume of the PTV respectively, and D_p_ is the prescription dose. Dose conformity was evaluated by calculating conformity number (CN) according to^39^:

(3) $$\mathrm{CN}\;=\frac{V_{\mathrm{PTV},\mathrm{RI}}}{V_{\mathrm{PTV}}}\times \frac{V_{\mathrm{PTV},\mathrm{RI}}}{V_{\mathrm{RI}}}$$

where V_PTV, RI_ is the volume of the PTV in cubic centimeters (cc) covered by the 100% isodose line (reference isodose), V_PTV_ is the volume of the PTV and V_RI_ is the volume of the reference isodose line (100%). Therefore, values of CN range within the interval 0 ≤ CN ≤ 0.95 (based on the plan normalization used), where 0 indicates a very poor conformity. The maximum dose (*D*_max_) was also recorded, defined as the greatest dose received by a volume of > = 0.03 cc of the structure. The near maximum dose (*D*_2%_) was also recorded, defined as the dose to 2% of the volume.

Finally, values of maximum (*D*_max_), mean (*D*_mean_) and median (*D*_median_) doses for all OARs were also recorded to for plan comparison. Integral dose (ID) received by the patient's body was also calculated since that is an important dosimetric parameter for evaluating potential reduction of patient exposure to radiation, especially in noncancer diseases.^40^, ^41^ For each patient, ID was calculated for both plans (conventional and 4pi optimized) according to^42^:

(4) $$\mathrm{ID}\;=D_{mean}\times \;V$$

where *D*_mean_ is the mean dose in gray (Gy) delivered to the volume V in liter (L).

To determine the significance of difference in these metrics between plans, a Wilcoxon signed rank test was performed for the patient population used in this study with a confidence level of 95% (*α* = 0.05). This assumes that data are paired and arise from the same population, and that each pair is randomly chosen, however does not require a normal probability distribution.

In order to calculate quantities of interest and expedite analysis, a separate stand‐alone ESAPI script was built allowing access to all calculated dosimetric data within the planning system. Therefore, after performing the VMAT optimization of both treatment plans, the application was run for extracting data of all patients and performing the statistical analysis described above.

## 3 RESULTS AND DISCUSSION

### 3.1 Sparing of noncardiac structures

Table 3 presents the *p*‐values obtained from the Wilcoxon signed‐rank test applied to maximum, mean and median doses received by each OAR in order to identify statistically significant differences between conventional and 4pi‐optimized trajectory plans. Although our analysis revealed differences between the two methodologies, both conventional and 4pi plans would be deemed acceptable for treatment by our clinicians due to the agreement between calculated dose metrics and recommended dose constraints presented in Table 1.

**TABLE 3:** *p*‐Values for maximum, mean and median doses to OARs (including cardiac structures) investigated in this study (*N* = 15 patients)

OAR	Maximum dose p‐value	Mean dose p‐value	Median dose p‐value
	Non‐cardiac OARs		
Esophagus	0.004	0.003	0.001
Spinal cord	0.003	0.001	0.001
Skin	0.140*	0.001	0.001
Liver	0.173*	0.001	0.001
Lungs	0.009	0.001	0.001
Stomach	0.125*	0.211*	0.001
Trachea	0.009	0.001	0.001
	Cardiac structures		
Aorta	0.532*	0.005	0.001
Right atrium	0.460*	0.053*	0.078*
Left atrium	0.005	0.496*	0.211*
Heart‐PTV	0.003	0.03	0.088*
Right ventricle	0.002	0.017	0.011
Left ventricle	0.005	0.004	0.003
IVC	0.955*	0.027	0.078*
SVC	0.570*	0.001	0.001
Pulmonary veins	0.047	0.256*	0.031
Pulmonary trunk	0.691*	0.001	0.001

*Not statistically significant for *p* > 0.05.

For all OARs outside of the heart differences in maximum doses between the two approaches were statistically significant, except for skin, liver and stomach. The greatest value of maximum dose for the skin was found to be 9.4 Gy, which is well below the recommended dose constraint of 26 Gy for one single fraction treatment shown in Table 1. Similarly, the volume of the liver receiving 9.1 Gy (V_9._1 Gy) for all patients was below 9 cc which is also much lower than the 700 cc recommended value.

For the stomach, one patient presented *D*_max_ values of 20 Gy and 27 Gy, respectively, for both conventional and 4pi trajectories. Due to the proximity of this OAR to the target this patient also showed the greatest loss of conformity (≈ 32%). This result shows that OARs dose sparing using the 4pi technique can be quite challenging for immediately proximal structures. Beam's‐eye‐view (BEV) overlap is minimized for the entire structure, and so the optimizer will determine trajectories with the least amount of overlap between the structures, regardless of that overlap's proximity to the target. A potential future improvement for larger structures would be for the optimizer to prioritize a subvolume of the OAR that is proximal to the target volume. All values of *D*_max_ for the stomach were below 12 Gy for the remaining patients. Although values of *D*_max_ for the stomach greater than the recommended constraint was observed for only one patient for both, 4pi and conventional trajectories, this result shows the importance of additional care when defining treatment plan priorities especially when the referred OAR is immediately proximal to the heart. As we stated above, no significant difference (*p* = 0.125) was observed between the two techniques in terms of maximum dose to the stomach (as well as to skin and liver) for all patients.

Differences in mean doses were found to be statistically significant for all OARs except the stomach, although mean dose values to this OAR were below 3.5 Gy for all patients when using the 4pi technique. Significant differences in median doses between the two plans were also observed for all OARs outside the heart.

**FIGURE 3:**
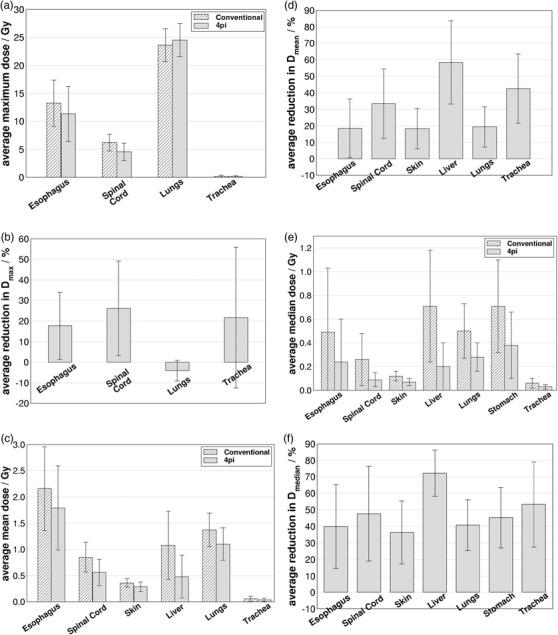
(a) Comparison of average (*N* = 15) maximum dose values for noncardiac OARs obtained with conventional and 4pi optimized trajectories that presented statistically significant differences (*p* < 0.05). Error bars represent the standard deviation of individual data values to the mean. (b) Average maximum dose reduction obtained with the 4pi optimized trajectory. Panels (c)–(f) show the same as in (a) and (b), but for mean and median doses, respectively

Figure 3 compares OARs doses averaged over the 15 patients that showed statistically significant differences (*p* < 0.05) between the conventional and 4pi optimized trajectory plans. We can see from Figure 3(a) that the 4pi optimized trajectory provided significant dose sparing in maximum dose to the esophagus (*p* = 0.004), spinal cord (*p* = 0.003) and trachea (*p* = 0.009). It should be noted, however, that maximum dose values for these OARs are much below the constraints suggested in Table 1. The improvement in dose sparing can also be visualized in Figure 3(b) which shows the average percent reduction in maximum dose to each OAR after trajectory optimization. Here, differences between maximum doses obtained with the two techniques for each patient were normalized to the value in the conventional plan before averaging across all patients. A negative value represents an increase for the referred parameter.

Figure 3(c) shows that significant improvement in dose sparing with the 4pi optimized plans was realized in terms of mean dose for esophagus (*p* = 0.03), spinal cord (*p* = 0.001), skin (*p* = 0.001), liver (*p* = 0.001), lungs (*p* = 0.001) and trachea (*p* = 0.001). Percent reduction in mean dose to those OARs were greater than 18% as shown in Figure 3(d). An improvement in dose sparing with percent reduction of greater than 20% in median dose (*p* = 0.001) was also found for all OARs outside the heart investigated in this work as shown in Figure 3e,f. Analysis of the averages for all OARs presented in Figure 3 across all patients shows that the implementation of an optimized trajectory provided lower dose values for all sensitive structures for both mean and median doses. For maximum doses, all OARs, but one, had improved sparing with the 4pi‐optimized trajectory plans compared to the conventional plans. Average maximum, mean and median doses were found to decrease by 21.8%, 31.8%, and 47.9% respectively. The one exception to the consistent finding of reduced sparing, over all metrics, was for the lungs, where a slight increase of maximum dose (*p* = 0.01) when using the optimized trajectory plan was observed. We note that the dose constraint for lungs is normally not with regard to the dose maximum as it is a parallel OAR.

A significant reduction (*p* = 0.001) of the integral dose received by the body resulted when using the 4pi optimized trajectories, which is likely related to reduced values of mean doses. Average values over entire population were 20.9 ± 3.8 GyL and 17.6 ± 4.3 GyL for the conventional and 4pi trajectories respectively.

**FIGURE 4:**
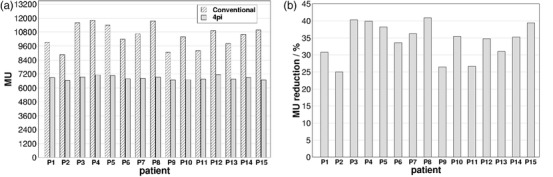
(a) Comparison of total MUs obtained for the two treatment plans (conventional and 4pi optimized) for all 15 patients. In (b), the reduction in total MUs obtained with the 4pi trajectories

Figure 4(a) compares the total MUs for both treatment plans for each patient with average values of 10 483 ± 970 MUs and 6845 ± 160 for the conventional and 4pi trajectories respectively. A significant reduction (*p* = 0.001) was observed on the amount of MUs when using the 4pi trajectories with an average of 34.3% ± 5.3 % as it is shown in Figure 4(b). That represents an important advantage of the 4pi method since it can reduce the treatment time. Despite changing a single 360^o^ arc at a single couch angle to multiple arcs at multiple couch angles may increase the total treatment time due to the time required to move the couch, that increase may not be significant especially considering the significant reduction in the amount of MUs delivered with the optimized arc trajectories.

**FIGURE 5:**
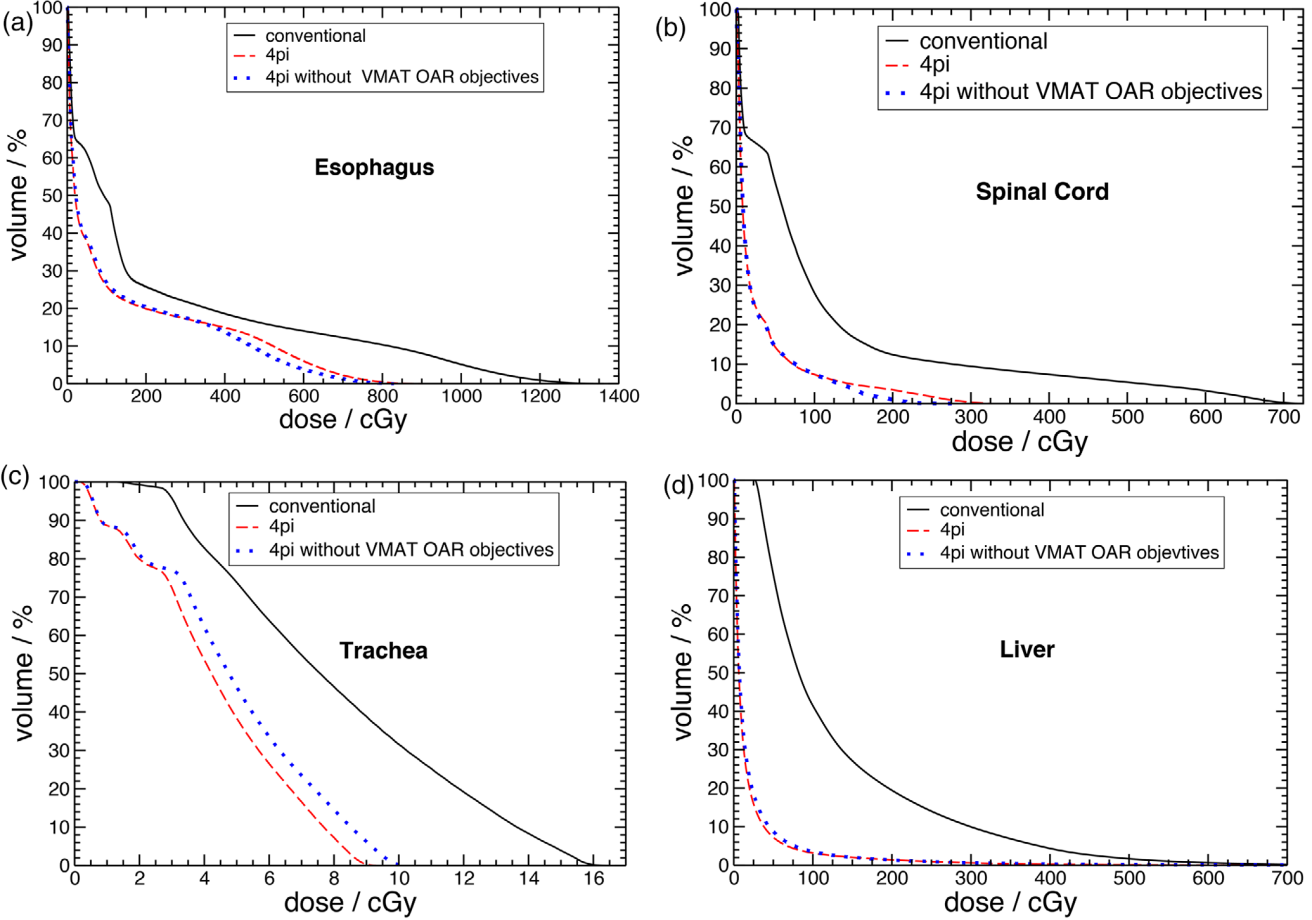
DVHs for (a) esophagus, (b) spinal cord, (c) trachea, and (d) liver for one selected patient

As demonstrated by MacDonald and Thomas,^33^ it is important to confirm that improvements in dose sparing arise from the 4pi‐optimized trajectories, and are not simply provided by the subsequent VMAT optimization. To verify this, one patient was chosen at random and its 4pi‐optimized trajectory plan was reoptimized after omitting all OAR dose objectives (although the automatic NTO was maintained). In this way the VMAT algorithm is allowed to prioritize only the PTV coverage and homogeneity. Figure 5 gives the comparison between dose volume histograms (DVHs) obtained for the conventional trajectory plan, the 4pi‐optimized trajectory plan using the same VMAT objectives for the OARs, and the optimized trajectory plan without OAR objectives. This comparison is shown for esophagus (Figure 5a), spinal cord (Figure 5b), trachea (Figure 5c) and liver (Figure 5d) for one selected patient. As MacDonald and Thomas^33^ pointed out in their study, it is reasonable to extrapolate the result to other test‐patients with similar anatomical arrangements if the optimized trajectories for those other patients involve a similar degree of overlap minimization. This demonstrates the similarity of DVHs with and without OAR dose objectives, as well as the significant improvement of dose metrics relative to the conventional plan. This also underlines a previously‐reported^33^ characteristic of 4pi‐optimized plans, that is, when trajectories are selected to avoid overlap with OARs, the VMAT optimization becomes less important with regard to sparing. Even for the liver where maximum doses did not present significant differences, Figure 5(d) shows significant improvement in dose sparing in terms of mean (and median) dose provided by the optimized trajectory plans. Similar results were found for the remaining patients and other OARs and are shown in Table 4 by comparison of the dose to 50% of the volume (*D*_50%_) of the OAR. Average values of *D*_50%_ over all 15 patients for the plans with and without OAR dose objectives are closer to each other and lower than the values found for the conventional plan. Except for IVC and pulmonary veins where there was not significant statistical difference between the 4pi plans and the conventional one, all OARs presented lower values of *D*_50%_ when using optimized trajectories. That also confirms the improvement in dose sparing provided by the 4pi trajectories.

**TABLE 4:** Average values of *D*_50%_ (dose to 50% volume) obtained with conventional, 4pi and 4pi without VMAT objectives plans for OARs of all 15 patients. *p*‐Values shown between parenthesis in third and fourth columns indicate if there was statistical significant difference between each 4pi plan (with and without VMAT objecftives) and the conventional one

OAR	D_50%_ (cGy)		
	Conventional	4pi	4pi without VMAT objectives
Esophagus	48.7 ± 53.8	23.9 ± 35.7 (*p* = 0.001)	27.2 ± 39.9 7 (*p* = 0.009)
Spinal cord	26.5 ± 22.2	9.4 ± 5.5 (*p* = 0.001)	10.4 ± 6.1 (*p* = 0.005)
Skin	11.7 ± 4.5	7.1 ± 2.7 (*p* = 0.001)	8.1 ±3.1 (*p* = 0.001)
Liver	70.68 ± 47.43	20.1 ± 20.3 (*p* = 0.001)	20.3 ± 18.6 (*p* = 0.001)
Lungs	50.4 ± 22.6	28.3 ± 11.7 (*p* = 0.001)	32.1 ± 12.9 (*p* = 0.001)
Stomach	73.3 ± 39.4	38.2 ± 27.7 (*p* = 0.001)	44.4 ± 34.0 (*p* = 0.001)
Trachea	5.7 ± 4.5	2.7 ± 2.2 (*p* = 0.001)	3.1 ± 2.6 (*p* = 0.001)
Aorta	45.2 ± 29.4	18.9 ± 7.5 (*p* = 0.001)	22.4 ± 9.4 (*p* = 0.003)
IVC	308.0 ± 161.1	260.5 ± 193.8 (*p* = 0.078)*	319.2 ± 291.5 (*p* = 0.650)*
SVC	20.5 ± 18.7	9.1 ± 4.0 (*p* = 0.001)	11.0 ± 5.1 (*p* = 0.020)
Pulmonary veins	82.8 ± 41.9	61.8 ± 59.7 (*p* = 0.031)	73.0 ± 68.0 (*p* = 0.211)*
Pulmonary trunk	49.1 ± 35.2	22.8 ± 12.9 (*p* = 0.001)	26.9 ± 13.0 (*p* = 0.020)

*Not statistically significant for *p* > 0.05.

### 3.2 Sparing of cardiac structures

**FIGURE 6:**
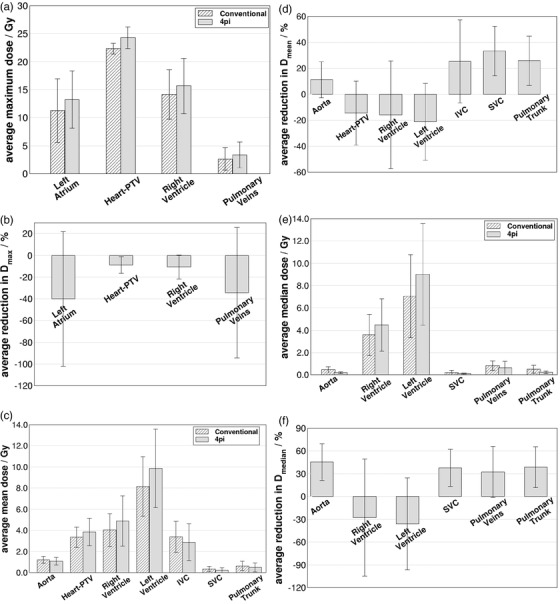
(a) Comparison of average (*N* = 15) maximum dose values obtained for cardiac structures with conventional and optimized trajectories that presented statistically significant differences (*p* < 0.05). Error bars represent the standard deviation of individual data values to the mean. (b) Average (over all patients) maximum dose reduction obtained with the optimized trajectory. Panels (c)–(f) show the same as in (a) and (b), but for mean and median doses, respectively

Table 3 shows that most cardiac structures presented significant differences between conventional and 4pi‐optimized trajectory plans especially in terms of mean and median dose. Figure 6 shows the comparison between those two approaches with significant differences in terms of dose to cardiac structures. Comparison of average maximum doses over all patients for left atrium (*p* = 0.005), heart‐PTV (*p* = 0.003), right ventricle (*p* = 0.002), left ventricle (*p* = 0.005) and pulmonary veins (*p* = 0.047) are shown in Figure 6(a). This indicates, in general, higher maximum doses for these OARs when using the 4pi‐optimized trajectories, with the relative change in maximum dose given in Figure 6(b). While the 4pi optimization method is effective at sparing more distant OARs, sparing immediately proximal structures remains challenging, and can even be reduced when more distant OARs affect the optimization. Since the PTV is located (usually) within the ventricular muscle, the optimized trajectory provides little advantage for sparing maximum dose for proximal OARs such as heart chambers and vasculature. On the other hand, it is important to point it out that the VMAT optimization was run with one single interaction with the optimizer to make a reasonable comparison between conventional and 4pi trajectories, as described above. In this sense, it is possible that maximum dose to cardiac structures may be reduced by forcing the optimizer to improve dose sparing on those referred structures.

Similarly, higher mean dose values were also found for heart‐PTV (*p* = 0.03), right ventricle (*p* = 0.017) and left ventricle (*p* = 0.004) as shown in Figure 6(c),(d). On the other hand, the optimized trajectory did provide improvements in sparing mean doses to aorta (*p* = 0.005), IVC (*p* = 0.027), SVC (*p* = 0.001) and pulmonary trunk (*p* = 0.001). Median dose values were also lowered by the 4pi approach for aorta (*p* = 0.001), SVC (*p* = 0.001), pulmonary veins (*p* = 0.031) and pulmonary trunk (*p* = 0.001) as shown in Figure 6(e),(f). However, an increasing in median doses to right ventricle (*p* = 0.011) and left ventricle (*p* = 0.003) were also observed with the optimized trajectory.

The increase in maximum dose values for structures near the target can be understood by considering that the 4pi method optimizes arc trajectories, which may not have a large impact with regard to shaping the dose distribution immediately proximal to the PTV. It is important to note that for most heart substructure OARs evaluated in this study, dose values achieved remain below recommended values in the literature^19^ such as those reported for aorta (*D*_max_ ≤ 20 Gy), heart‐PTV (*D*_50%_ ≤ 5 Gy) and SVC (*D*_50%_ ≤ 0.6 Gy). Our results show a maximum value of 17.3 Gy for the maximum dose (*D*_max_) to the aorta. Maximum values of dose delivered to 50% of the volume of the organ (*D*_50%_) were found to be 4.3 Gy and 0.17 Gy to the heart‐PTV and SVC respectively.

Although some cardiac structures presented in Figure 5 were found to increase dose values when examining the maximum dose, values reported in this study are not unexpected considering the target is located within the heart. Furthermore, lower maximum dose values for cardiac structures can still be pursued during the treatment planning according to specific clinical priorities and goals.

### 3.3 PTV coverage

There was no statistically significant difference (*p* = 0.650) with regard to PTV metrics in maximum dose using both techniques. Averaged over all 15 target volumes, values of *D*_max_ were 31.6 ± 0.9 Gy and 31.6 ± 1.0 Gy for conventional and 4pi‐optimized trajectory plans, respectively. Similarly, the near‐maximum dose metric (*D*_2%_) did not present any significant difference (*p* = 0.334) between the two approaches. Averages were *D*_2%_ = 30.8 ± 0.7 Gy and *D*_2%_ = 30.9 ± 0.8 Gy for conventional and 4pi‐optimized trajectory plans respectively, which is in fair agreement with the limitation of 32.5 Gy suggested by Blanck et al.^20^ for this parameter.

Similarly, dose homogeneity was not found to be statistically different (*p* = 0.156) when using the conventional or the 4pi‐optimized approach. The average homogeneity index, as defined in Equration 2, was HI = 21.5% ± 2.4% for the conventional trajectory plan and HI = 22.2% ± 3.1% for the 4pi‐optimized one, respectively. Combined with the results for *D*_max_ and *D*_2%_, this suggests that using a 4pi‐optimized trajectory will not compromise PTV dose homogeneity metrics compared to conventional approaches.

Conversely, the conformity number as defined in Equation (3) was found to decrease (*p* = 0.001) when using the 4pi‐optimized trajectory with average values (over all PTVs) going from CN = 0.86 ± 0.05 (conventional) to CN = 0.77 ± 0.09 (4pi‐optimized). Despite providing dose sparing to OARs, 4pi trajectory optimization is not expected to improve dose conformity to PTV since that is not taking into consideration in the geometric optimization code. Similarly, minimum (*D*_100%_) and near‐minimum (D_98%_) doses were found to decrease (*p*‐values of 0.003 and 0.005 respectively) when using the 4pi trajectories. Average values of *D*_100%_ were 20.4 ± 1.1 Gy for conventional trajectory and 19.2 ± 1.4 Gy for the 4pi one. For D_98%_ average values were 24.3 ± 0.3 Gy and 24.1 ± 0.3 Gy for conventional and 4pi trajectories, respectively.

When VMAT optimization is performed for the randomly selected patient mentioned in Section 3.1, using the same objectives, the conventional and 4pi‐optimized trajectories gave similar results in terms of target dose homogeneity and conformality, on average. For the conventional plan, HI = 22.17% and conformation number was CN = 0.86, while for the 4pi‐optimized plan, HI = 22.26% and CN = 0.81. However, after removing dose objectives in the VMAT optimization for the 4pi‐optimized trajectory these values are altered to HI = 13.4% and CN = 0.84. This is expected given that without OAR objectives, the VMAT optimization is allowed to prioritize the PTV, including dose homogeneity without being constrained by OAR sparing.

### 3.4 Limitations

One of the main limitations of the present study was the fact that the CT images used from the public database do not represent real cases (lesions) of ventricular tachycardia. Hearts with scar related VT are geometrically different from hearts without VT since the former ones have scars.^42^, ^43^ However, since targets were delineated in this study to be realistic with direct reference to actual case studies,^19^ those differences are expected to be accounted for in the PTV definition.

Additionally, the impact of combined effect of respiratory and cardiac motion could not be assessed in this current study since that would demand the availability of 4D‐CT images. The use of additional image modalities to improve organs contouring were not possible either since there was no other type of image for the patients used in this study.

## 4 CONCLUSIONS

In this work, we investigated potential advantages of using 4pi‐optimized arc trajectories in the context of VT‐SBRT in order to improve dose sparing to OARs around the target. Our results indicate that significant dose reduction to a range of relevant OARs may be realized. Analysis of integral dose absorbed in the whole patient body also reveals a potential benefit for reducing the risk of toxicities for health tissues away from the treatment field. Additionally 4pi plans were found to provide a potential reduction in treatment time delivering a smaller amount of MUs compared to the conventional trajectory.

Although the sparing of immediately proximal cardiac sub‐structures was not improved with regard to maximum dose, improvements were seen with regard to median and mean dose, and all dose metrics remained below those reported or recommended in the literature to date. Furthermore, reduction in dose metrics can still be achieved for individual VMAT plans by forcing the optimizer to spare dose to specific structures while reaching desired coverage according to clinical goals and priorities. Since dose constraints in the context of VT‐SBRT are still under investigation, dose to relevant OARs should be kept as low as reasonably achievable, and the 4pi‐optimization of arc trajectories is sufficiently flexible that the priorities of OARs may be adjusted as clinical knowledge evolves.

## CONFLICT OF INTERESTS

R. Lee MacDonald reports grants from Brainlab AG, outside the submitted work; In addition, he has a patent US patent: US 10, 441, 813 B2; with royalties paid to Brainlab AG. Alasdair Syme reports grants from Atlantic Canada Oppurtunities Agency ‐ Atlantic Innovation Fund, outside the submitted work. Christopher G. Thomas reports grants from Atlantic Canada Oppurtunities Agency ‐ Atlantic Innovation Fund, grants from Brainlab AG, outside the submitted work; In addition, he has a patent "Method and System for Cancer Treatment with Radiation" with royalties paid to Brainlab AG. James L. Robar reports grants from Varian Medical Systems, other from Adaptiiv Medical Technologies, outside the submitted work. The other authors declare no conflict of interest.

## AUTHOR CONTRIBUTIONS

C.Q.M. Reis: *Countoring, treatment planning, data analysis, software development (data analysis), and manuscript preparation*.

B. Little: *Software development (4pi algorithm) and critical review of the manuscript*.

R. Lee MacDonald: *Software development (4pi algorithm) and critical review of the manuscript*.

Alasdair Syme: *Software development (4pi algorithm) and critical review of the manuscript*.

Christopher G. Thomas: *Software development (4pi algorithm) and critical review of the manuscript*.

James L. Robar: *Study conception, software development (data analysis), analysis, contour review and manuscript review and supervised the project*.

All authors discussed the results and contributed to the manuscript.

## Data Availability

The data that support the findings of this study are openly available in [DRYAD] at [https://datadryad.org/stash/dataset/ https://doi.org/10.5061/dryad.mj76c], ref. [37], and in [Cancer Imaging Archive ‐ CT Lymph Nodes ‐ Mediastinum] at [https://www.cancerimagingarchive.net/collections/], ref. [36]
